# Gender differences in spontaneous adverse event reports associated with zolpidem in South Korea, 2015–2019

**DOI:** 10.3389/fphar.2023.1256245

**Published:** 2023-11-09

**Authors:** Kyung-In Joung

**Affiliations:** School of AI Healthcare, College of Integrated Health Science, CHA University, Pocheon, Republic of Korea

**Keywords:** zolpidem, adverse event, gender difference, somnambulism, sleepwalking, voluntary adverse drug events reporting system

## Abstract

**Study objectives:** While zolpidem is considered as an example of a gender effect on drug response, there is insufficient evidence to reach a consensus. This study aimed to investigate gender differences in adverse events (AEs) of zolpidem.

**Methods:** We estimated the difference between the reporting odds ratios (RORs) calculated in gender subgroups for the AEs signals detected in data mining using 2015–2019 Korea voluntary adverse drug events reporting system (KAERS) data. Different reporting risk by gender was evaluated by using the log RORs being significantly different by gender at the 5% significance level and the 95% confidence intervals of the gender ROR.

**Results:** A total of 94 AE signals were detected. Among these, 35 signals showed significant disparities by gender at the 5% level or were detected only in one gender. When categorized by similarity of AEs, parasomnia including somnambulism and paroniria, and cardiovascular disorders including coronary thrombosis had higher reporting risks in women. Men were more likely to report cognitive disorders such as delirium, insomnia related disorders, and movement disorders. Among all AEs with gender differences in reporting risk, the difference in somnambulism was the most consistent and substantial.

**Conclusion:** For several AEs associated with zolpidem, gender-based reporting disparities were evident. Notably, women exhibited a higher susbeptibility to somnambulism, potentially serious adverse effects of zolpidem. This underscores the need for further investigation into the underlying factors influencing these gender-specific reporting patterns.

## Introduction

Zolpidem is a major hypnotic agent that selectively targets the γ-aminobutyric acid type A (GABAA) receptor. Due to the short acting effect and general tolerability, it has been a preferred choice among prescribers and patients for treating insomnia (Olson, 2008). With the significant increase in the use of zolpidem, post-marketing studies and case reports have indicated rare but associated sleep-related complex behaviors, making it a key warning for this medication (Daley et al., 2011). Another specific issue for zolpidem is for its sex-related differences in pharmacokinetic and pharmacodynamic parameters leading to the US FDA issuing recommendations in 2013 to lower the initial dosage of Zolpidem for women (Communication FaDADS, 2023). For women, the FDA warned to reduce initial dosage of zolpidem to 5 mg of immediate release (IR) tablets, or 6.25 mg of modified release (MR) tablets (FDA FaDA, 2023).

However, there is an argument that these regulatory actions lack a confirmative clinical evidence (Greenblatt et al., 2000; Greenblatt et al., 2019; Yoon et al., 2021). According to a recent study evaluating gender effects on zolpidem through an analysis of data from an existing study, no gender-related difference in clinical efficacy or adverse reactions was demonstrated, although lower clearance of zolpidem in women than in men was apparent which could not be explained by body weight (Greenblatt et al., 2019). So far, no other country outside the United States, including Korea, has taken such a measure to reduce the initial dose of zolpidem in women.

Gender is rarely taken into account in the majority of mental health studies (Howard et al., 2017). For instance, a study found that less than 1% reported intention to analyze by gender among 768 trials of treatments for depression on ClinicalTrials.gov (Weinberger et al., 2010). Despite having several drawbacks, spontaneous adverse event (AE) reporting data is a valuable real-world data source for pharmacovigilance investigations (World Health Organization, 2002; Palleria et al., 2013). Even in research that used these data, gender was rarely taken into account and to our knowledge, no study using these data to examine the adverse effects of zolpidem focused on gender differences exists (BEN‐HAMOU et al., 2011; Wong et al., 2017; Greenblatt et al., 2019).

As such, although zolpidem is cited as an example of a gender effect on drug response, there is no consensus in both regulatory authorities worldwide and healthcare professionals due to insufficient scientific evidence. This study aimed to explore gender differences in adverse effects related to zolpidem using Korea’s voluntary adverse events reporting data.

## Methods

### Data source

The Korea Adverse Event Reporting System (KAERS) was retrospectively observed as the data source for the analysis, which covered the period between January 2015 and December 2019. The Korea Institute of Drug Safety and Risk Management (KIDS) established the automated AE reporting system known as KAERS in 2012. Both databases contain voluntarily submitted AE reports from consumers, healthcare professionals, 27 local pharmacovigilance centers, and marketing authorization holders, most of which are pharmaceutical firms. Reports from all types of reporters were included in the analysis. Each case contains data on the patient’s age, sex, administration date of zolpidem, type, and symptom of AEs, and patient outcomes without identifying any particular individuals. The international drug monitoring program operated by the WHO-Uppsala Monitoring Center is compatible with the KAERS database. The Anatomical Therapeutic Chemical Classification System (ATC Code) was utilized to record the drug names, and the World Health Organization- Adverse Reaction Terminology (WHO-ART)’s preferred terms (PTs) were use to code the adverse events (AEs). The KIDS (https://open.drugsafe.or.kr/original/invitation.jsp) websites host the KAERS datasets (Shin et al., 2009; Joung et al., 2020a).

### Exposure definition and data mining for signal detection

Exposure was defined as reported zolpidem use. Disproportionality analysis methods including proportional reporting ratios (PRR) (Evans et al., 2001), reporting odds ratios (ROR) (Rothman et al., 2004), and Information component (IC) of Bayesian confidence propagation neural network (BCPNN) (Norén et al., 2008) were used to identify adverse reaction signals of zolpidem in KAERS. Calculations of measures of disproportionality are primarily based on a two-by-two contingency table (van Puijenbroek et al., 2002). In brief, PRR is the proportion of AEs in zolpidem divided by the fraction of specific AEs in all other drugs, and the criteria for conforming to the signal are PRR ≥ 2, chi-square ≥ 4, and the number of cases with AEs ≥ 3, and the ROR The formula is (A/C)/(B/D), and the criteria for signal are ROR ≥ 2, chi-square ≥ 4, and the number of cases with AEs ≥ 3. The IC is the logarithmic value of the probability of using a certain drug multiplied by the probability of the occurrence of a specific AE if the use of that drug and the occurrence of the particular AE are independent of each other. The formula for the calculation of IC is and the criterion is when the lower limit of the 95% confidence interval is higher than 0. In this study, AEs that satisfied all three criteria (PRR, ROR, and IC) were defined as signals.

We investigated the ROR of adverse drug reactions (ADRs) grouped by ADR type, which can be difficult for reporters to distinguish due to their similarity, or can be grouped together by a common characteristic, such as parasomnia (Bjorvatn et al., 2010), which include both paroniria and somnambulism, and the cardiovascular disorder group, which includes eight diseases such as cardiac failure, heart disorder, and coronary thrombosis. Because the PTs are highly specific and in some cases, one PT may belong to a sub-group of another PT, making them not mutually exclusive, we considered it more rational to investigate the RORs by ADR category rather than individual ADRs of PT level.

### Verification of gender differences and statistical analysis

To explore the gender differences, each analysis was performed separately by gender. The *t*-test and Chi-square test were applied for continuous variables and categorical variables, respectively to examine the differences in the basic demographic and AE reporting data by gender. Serious AEs refers to any of the following: 1) death or a life-threatening condition, 2) hospitalization or prolongation of existing hospitalization, 3) persistent or significant disability/incapacity, 4) congenital anomaly/birth defect, 5) any other medically important condition that requires medical intervention, such as drug dependence or abuse, or a blood disorder, etc.

For each signal detected, we calculated the frequency and ROR with 95% confidence intervals for each gender subgroup. The difference between the two odds ratios was estimated as the difference between the logarithms of the two RORs. We evaluated different reporting risks by gender using the log reporting odds ratios (RORs), with statistical significance determined at the 5% level, and the 95% confidence intervals of the gender ROR were also considered.

In a secondary analysis, we defined control group as patients exposed to benzodiazepine anxiolytic/hypnotic drugs, then the RORs were calculated. We expected the size (ROR) of signals detected in this analysis to generally be smaller than those in the primary analysis due to the similar mechanisms of action on the nervous system. Therefore, the number of signals detected was anticipated to decrease significantly. However, if we detected gender differences in the signals and reporting risk in this analysis, it would support the robustness of the primary study results. Benzodiazepine derivatives included all drugs in the WHO ATC N05BA category.

In another secondary analysis, RORs were calculated by gender for ADR categories only for suspected drugs. In KAERS, “suspected drugs” are drugs suspected to have caused the adverse reaction in question, while other drugs are classified as “concomitant drugs.”

All data were analyzed using the SAS statistical application program (Version 9.4, SAS Institute Inc., NC, United States).

## Results

The dataset consisted of 1,016,161 reports, with 599,311 reports from women and 416,850 from men. The number of drug-adverse event combinations was 3,524,587, with 11,341 AE reports associated with zolpidem, 5,791 occurring in women and 5,550 in men. In the reports containing zolpidem, 476 types of AEs were observed in women, and 468 in men. Out of the 2,442 reports that provided information on the daily dosage of zolpidem, there was no significant difference between women and men in the administered dosage, which was 8.55 and 8.54 mg, respectively. Among all reports, the proportion of serious AEs was lower in women than in men (7.37% vs. 10.28%), while the proportion of serious AEs associated with zolpidem use was similar between women and men (25.20% vs. 24.49%). The majority of reporters (96.49%) were healthcare professionals, such as doctors, pharmacists, and nurses (Table 1).

**TABLE 1:** Characteristics of zolpidem users whose adverse events were reported and their reports to the Korea Adverse Event Reporting System (KAERS), 2015–2019.

	Overall	Women	Men	*p* value
Age				<0.0001
Mean (SD)	54.14 (18.92)	53.84 (18.41)	54.56 (19.65)	
0–19	56,655 (5.58)	25,948 (4.33)	30,707 (7.37)	
20–39	159,198 (15.67)	105,485 (17.6)	53,713 (12.89)	
40–64	471,060 (46.36)	284,191 (47.42)	186,869 (44.83)	
≥65	329,248 (32.4)	183,687 (30.65)	145,561 (34.92)	
Number of reports	1,016,161 (100.0)	599,311 (59.0)	416,850 (41.0%)	<0.001
2015	165,973 (16.33)	95,675 (15.96)	70,298 (16.86)	
2016	195,773 (19.27)	115,672 (19.3)	80,101 (19.22)	
2017	219,073 (21.56)	129,762 (21.65)	89,311 (21.43)	
2018	218,250 (21.48)	129,092 (21.54)	89,158 (21.39)	
2019	217,092 (21.36)	129,110 (21.54)	87,982 (21.11)	
Number of AE types found in the reports containing zolpidem	621	476	468	<0.001
Number of AE types found in the reports containing all drugs other than zolpidem	1736	2024	1,518	
Average prescribed amount per dose, mg (SD)	8.54 (2.79)	8.55 (2.38)	8.54 (2.78)	>0.05
	N = 2,442	N = 1,222	N = 1,220	
Type of reporter				>0.05
Physician, Pharmacist, Nurse	10,563 (96.49)	5,374 (96.31)	5,189 (96.60)	
Consumer	289 (2.64)	165 (2.96)	124 (2.31)	
Others (other medical professional, lawyer)	95 (0.87)	41 (0.73)	54 (1.01)	
Serious adverse events (n, %)	2,838 (25.02)	1,479 (25.54)	1,359 (24.49)	>0.05
Death or life threatening (n, %)	258 (2.27)	103 (1.78)	155 (2.79)	<0.001
Suspected AEs	228	189	158	>0.05

Abbreviations: AE, adverse event; SD, standard deviation.

In total, 94 PT signals were detected. Supplementary Table S1 presents all signals detected in the overall population, their number of cases in zolpidem users, and the RORs (95% CI) by gender. Delirium was the most frequently reported AE, with 333 and 691 cases in women and men, respectively. Out of the 94 PTs, 14 PTs exhibited differences in ROR at the 5% significance level between men and women, with AE reports present in both genders. These PTs are presented in Table 2. Of these, somnambulism had a significantly higher ROR in women than in men, with non-overlapping 95% CIs and a much higher frequency of reporting in women (42 out of a total of 56 reports) compared to men. PTs for which the ROR was higher in men than in women included delirium, hyperkinesia, anxiety, and depression.

**TABLE 2:** Adverse events signals of zolpidem reported in both men and women, with significant gender differences in ROR.

No	Adverse event	Women		Men		Difference^a^	*p* value
		No. of cases	ROR^a^ (95% CI)	No. of cases	ROR (95% CI)		
1	Thrombosis coronary	4	12.34 (4.71–32.34)	1	1.02 (0.14–7.10)	2.51	0.026
2	Respiratory depression	3	8.93 (2.92–27.31)	2	1.39 (0.35–5.54)	1.86	0.043
3	Neuralgia	11	5.19 (2.89–9.34)	2	1.11 (0.28–4.44)	1.54	0.046
4	Infusion site reaction	10	3.37 (1.82–6.25)	3	0.89 (0.29–2.77)	1.33	0.045
5	Psychosis	7	27.90 (13.68–56.9)	4	7.56 (2.87–19.89)	1.31	0.042
6	Somnambulism	42	150.85 (117.06–194.39)	14	64.52 (40.23–103.48)	0.85	0.015
7	Speech disorder	21	7.18 (4.7–10.98)	14	3.44 (2.05–5.80)	0.73	0.035
8	Delirium	333	55.88 (50.39–61.97)	691	67.70 (62.92–72.84)	−0.19	0.010
9	Insomnia	309	4.16 (3.71–4.66)	253	5.17 (4.56–5.86)	−0.22	0.013
10	Hyperkinesia	52	17.31 (13.26–22.60)	95	29.57 (24.38–35.87)	−0.53	0.003
11	Anxiety	47	3.47 (2.61–4.61)	71	5.90 (4.68–7.44)	−0.54	0.005
12	Depression	24	2.37 (1.59–3.53)	37	5.09 (3.70–7.02)	−0.77	0.004
13	Aggressive reaction	2	1.90 (0.48–7.56)	11	8.66 (4.84–15.51)	−1.52	0.049
14	Inappropriate schedule of drug administration	1	0.47 (0.07–3.31)	8	3.81 (1.91–7.58)	−2.10	0.048

Abbreviations: ROR, reporting odds ratio; 95% CI, 95% confidence interval.

^a^ The difference was calculated by subtracting the log odds ratios in men from the log odds ratio in women.

Table 3 presents the PTs that are reported only in one gender. Anal ulcer had the highest reporting frequency with an ROR (95% CI) of 13.82 (7.74–24.68) in women. Tolerance was reported only in women, with a very high ROR of 54.81 (22.06–136.2) in women. Hepatic cirrhosis (nine cases) and vein varicose (seven cases) were reported only in men.

**TABLE 3:** Adverse events signals reported only one gender among zolpidem users.

No.	Preferred term	Women		Men	
		No. of cases	ROR (95% CI)	No. of cases	ROR (95% CI)
1	Anal ulcer	11	13.82 (7.74–24.68)	0	-
2	Lactation non-puerperal	7	3.23 (1.54–6.75)	0	-
3	Bowel motility disorder	7	11.76 (5.67–24.37)	0	-
4	Amenorrhoea	6	4.59 (2.07–10.16)	0	-
5	Atherosclerosis	4	8.46 (3.21–22.28)	0	-
6	Myocarditis	4	9.26 (3.52–24.36)	0	-
7	Tolerance	4	54.81 (22.06–136.2)	0	-
8	Leukaemia lymphocytic	3	8.63 (2.82–26.41)	0	-
9	Varicella	3	22.34 (7.46–66.88)	0	-
10	Wound dehiscence	3	10.27 (3.37–31.34)	0	-
11	Pain axillary	3	8.22 (2.69–25.15)	0	-
12	Hepatic cirrhosis	0	-	9	7.1 (3.72–13.55)
13	Acrodynia	0	-	7	13.91 (6.75–28.67)
14	Vein varicose	0	-	6	4.59 (2.07–10.15)
15	Skin depigmentation	0	-	4	10.32 (3.94–27.02)
16	Oculomotor nerve paralysis	0	-	4	13.63 (5.23–35.49)
17	Hepatitis cholestatic	0	-	4	14.72 (5.66–38.26)
18	Artery malformation	0	-	4	9.05 (3.45–23.75)
19	Retinal detachment	0	-	4	3.49 (1.32–9.25)
20	Decubitus ulcer	0	-	4	4.7 (1.78–12.42)
21	Haemangioma acquired	0	-	3	8.04 (2.63–24.53)

Abbreviations: ROR, reporting odds ratio; 95% CI, 95% confidence interval.

When the PTs were categorized as shown in Table 4, there were gender differences in six out of the 11 categories. Parasomnia, speech disorders, and cardiovascular disorders had higher RORs in women, while insomnia-related disorders, cognitive disorders, and movement disorders (marginally significant, *p* = 0.057) had higher RORs in men. Parasomnia showed the largest gender difference, with a higher ROR in women than in men (Table 5).

**TABLE 4:** Adverse event categories of interest and preferred terms defining cases.

	Category	Preferred terms included
1	Cognitive disorders	Delirium, amnesia, dementia, cognitive disorders, stupor
2	Insomnia related disorders	Sleep disorder, insomnia
3	Parasomnia	Somnambulism, paroniria
4	Speech disorders	Aphasia, speech disorder, communication disorder
5	Cardiovascular disorders	Cardiac failure, heart disorder, cardiomyopathy, myocarditis, thrombosis coronary, tachycardia supraventricular, tachycardia ventricular, ECG abnormal specific
6	Movement disorders	Extrapyramidal disorder, dyskinesia, gait abnormal, hemiparesis, hyperkinesia
7	Misuse	Inappropriate schedule of drug administration, medication error related problems, drug abuse, drug dependence, tolerance
8	Unexpected or increased response	Unexpected therapeutic effect, therapeutic response increased
9	Ocular disorders	Oculogyric crisis, oculomotor nerve paralysis, diplopia, xerophthalinia, retinal detachment
10	Dental disorders	Toothache, peridontal disorder
11	Otitis	Otitis externa, otitis media chronic

**TABLE 5:** Frequencies of categorized adverse events by gender, and its difference of women to men.

No.	Category	Women		Men		Difference^a^	*p* value
		No. of cases	ROR (95% CI)	No. of cases	ROR (95% CI)		
1	Parasomnia	73	70.28 (51.93–86.78)	32	31.25 (22.48–43.45)	0.81	0.004
2	Speech disorders	29	7.21 (5.03–10.35)	21	3.75 (2.45–5.74)	0.65	0.021
3	Cardiovascular disorders	42	3.65 (2.70–4.94)	26	1.97 (1.34–2.90)	0.62	0.010
4	Insomnia related disorders	351	4.50 (4.05–5.01)	302	5.82 (5.19–6.53)	−0.26	0.002
5	Cognitive disorders	468	24.50 (22.35–26.86)	841	37.34 (34.81–40.05)	−0.42	<0.001
6	Movement disorders	89	7.41 (6.02–9.12)	133	9.68 (8.17–11.46)	−0.27	0.057
7	Dental disorders	7	2.95 (1.41–6.16)	9	2.45 (1.28–4.69)	−2.67	>0.05
8	Otitis	5	3.71 (1.55–8.87)	3	2.75 (0.89–8.50)	−0.30	>0.05
9	Ocular disorders	18	3.49 (2.21–5.53)	12	2.15 (1.22–3.78)	−0.48	>0.05
10	Misuse	28	8.46 (5.86–12.21)	26	8.24 (5.64–12.05)	0.026	>0.05
11	Unexpected or increased response	7	6.85 (3.29–14.28)	3	8.36 (2.74–25.51)	−0.20	>0.05

Abbreviations: ROR, reporting odds ratio; 95% CI, 95% confidence interval.

^a^ The difference was calculated by subtracting the log odds ratios in men from the log odds ratio in women.

In a secondary analysis that limited the non-exposed control group to users of benzodiazepine derivatives, six AEs including cardiac failure and somnambulism were particularly higher in women, while insomnia and delirium had higher reporting risk in men. Cardiac failure was the most prominent PT level AE with predominance in women (difference in log ORs = 1.78, *p* value = 0.001). Somnambulism had the highest ROR among both genders, with higher reporting risk in women (difference in log ORs = 0.85, *p* value = 0.015) (Table 6). In the other secondary analysis focusing only on suspect cases, gender differences in AE categories were also found to be significant in parasomnia (difference in log ORs = 1.12, *p* value < 0.001) and cognitive disorders (difference in log ORs = −0.32, *p* value < 0.001) (Table 7).

**TABLE 6:** Secondary analysis I: frequencies of categorized adverse events by gender and its difference of women to men when non-exposed control group was confined as benzodiazepine derivate users.

No.	Adverse event	Women		Men		Difference^a^	*p* value
		No. of cases	ROR (95% CI)	No. of cases	ROR (95% CI)		
1	Cardiac failure	28	3.23 (2.23–4.68)	4	0.54 (0.20–1.45)	1.78	0.001
2	Neuralgia	11	5.19 (2.89–9.34)	2	1.11 (0.28–4.44)	1.54	0.046
3	Infusion site reaction	10	3.37 (1.82–6.25)	3	0.89 (0.29–2.77)	1.33	0.045
4	Hypoproteinaemia	12	2.49 (1.41–4.37)	7	0.75 (0.36–1.56)	1.20	0.012
5	Somnambulism	42	150.85 (117.06–194.39)	14	64.52 (40.23–103.48)	0.85	0.015
6	Paroniria	31	40.41 (28.93–56.43)	18	22.23 (14.24–34.70)	0.60	0.054
7	Delirium	333	55.88 (50.39–64.97)	691	67.70 (62.92–72.84)	−0.19	0.010
8	Insomnia	309	4.16 (3.71–4.66)	253	5.17 (4.56–5.86)	−0.22	0.013

Abbreviations: ROR, reporting odds ratio; 95% CI, 95% confidence interval.

^a^ The difference was calculated by subtracting the log odds ratios in men from the log odds ratio in women.

**TABLE 7:** Secondary analysis II: frequencies of categorized adverse events by gender, and its difference of women to men only for the suspected cases.

No.	Category	Women		Men		Difference^a^	*p* value
		No. of cases	ROR (95% CI)	No. of cases	ROR (95% CI)		
1	Parasomnia	72	152.24 (123.37–187.55)	32	49.45 (35.64–68.62)	1.12	<0.001
2	Cognitive disorders	389	146.40 (132.44–161.84)	746	202.48 (187.00–219.25)	−0.32	<0.001
3	Insomnia related disorders	119	3.75 (3.12–4.51)	92	4.82 (3.92–5.93)	−0.25	0.078
4	Cardiovascular disorders	1	0.63 (0.09–4.50)	1	0.51 (0.07–3.64)	0.21	0.88
5	Speech disorders	14	8.89 (5.28–14.98)	15	8.53 (5.17–14.10)	0.04	0.91

Abbreviations: ROR, reporting odds ratio; 95% CI, 95% confidence interval.

^a^ The difference was calculated by subtracting the log odds ratios in men from the log odds ratio in women.

## Discussion

This study aimed to investigate gender differences in AEs associated with zolpidem using voluntary AE reporting data in Korea. Due to potential differences in reporting behavior of adverse drug reactions between genders (Mosnier-Pudar et al., 2009; Holm et al., 2017), and the possibility of different numbers of zolpidem users between genders (Joung et al., 2020b), we estimated the difference between the RORs calculated in gender subgroups, rather than comparing reporting frequencies or rates. Our findings showed a similar AE signal in zolpidem users as previous studies, with most AEs related to the central nervous system. Compared to a previous study using the 1988–2015 KAERS data in 2018, our study detected significantly more signals (94 vs. 59) due to the establishment of a voluntary adverse drug events reporting system through the internet by the Korea Institute of Drug Safety and Risk Management in 2012 (Han et al., 2018).

When comparing by gender, we found that the distribution of AEs was different, with 38% of AEs in PT level (36 out of 94) having different reporting risks by gender. Somnambulism had a significantly higher ROR in women than in men, while delirium, hyperkinesia, anxiety, and depression were higher in men. Categorizing PTs according to the similarity of AEs revealed that parasomnia and cardiovascular disorders had a higher risk of reporting in women, while insomnia related disorders, cognitive disorders, and movement disorders (marginally, *p* = 0.057) were dominant in men. Parasomnia had the largest gender difference in ROR among all PT-level AEs.

### Somnambulance

Complex sleep behaviors, which are mainly induced by non-benzodiazepine hypnotics, are not clearly defined in terms of the types of behaviors involved, but common examples include sleep walking, sleep-related eating, sleep conversations, sleep sex, and driving (Harbourt et al., 2020). Although complex sleep behaviors are rare, identifying its risk factors is essential from a medical and public health perspective, as they can result in serious consequences such as self-harm, falls, attacks on others, or even criminal acts (Daley et al., 2011).

While, the WHO-ART classification may not be sufficient in identifying the specific symptoms of complex sleep behaviors, as it only lists somnambulism (sleepwalking) as a symptom, this study found that somnambulism was reported three times more frequently in women than in men, and had the largest gender difference in ROR among all PT-level AEs. Although there is limited research on whether the risk of complex sleep behaviors due to zolpidem is related to gender, previous case reports and a systematic review are consistent with our findings, suggesting that women may be more at risk (Dolder and Nelson, 2008; Cubała and Gabrielsson, 2014; Stallman et al., 2018). However, a case-control study in nonpsychotic patients (Chen et al., 2013) and a cross-sectional study in psychiatric outpatients (Chen et al., 2014) found that sex was not associated with the risk of complex sleep behaviors.

Although these examples are exceptional, a review of post-2000 literature has demonstrated that out of five cases of homicide related to zolpidem use among patients with mood or anxiety disorders (Westermeyer and Carr, 2020), three were committed by women and two by men (Daley et al., 2011; Paradis et al., 2012; Edinoff et al., 2021). To our knowledge, this study is the first to confirm, through a large-scale voluntary reporting system, that zolpidem-related somnambulism is more commonly reported in women. The sex differences in reporting somnambulism were consistently observed in both secondary investigations, which supports the robustness of the findings.

### Cardiovascular disorder

There have been several studies on the association between zolpidem use and cardiovascular or cerebrovascular risks (Huang et al., 2013; Lee et al., 2014; Hu et al., 2022), but the results are inconsistent, and no research appear to have taken gender into consideration. Some studies have found an increased risk of adverse cardiovascular events, such as atrial fibrillation (Hu et al., 2022) and stroke (Huang et al., 2013; Lee et al., 2014) in zolpidem users, while other studies have reported a decreased risk of stroke (Zhu et al., 2016) or cardiovascular risk (Kim et al., 2018). Currently, the drug label in the US includes rare cardiovascular adverse effects such as arrhythmia, myocardial infarction, and hypertension (DAILYMED, 2023), while Korea and the United Kingdom do not list any significant cardiovascular-related adverse effect (Center KPI, 2023; Electronic Medicines Compendium, 2023).

In our study, a total of 68 cases of cardiovascular AEs associated with zolpidem were reported, of which 42 cases were reported in women, and the gender difference in log ROR was significant at 0.62. Besides, although the frequencies were low, signals of coronary thrombosis and myocarditis were detected only in women. In the secondary analysis, which set users of benzodiazepine anxiolytics/hypnotics as the non-exposed control group, heart failure showed the largest gender difference in ROR which is in line with the primary analysis. On the contrary, only two cases of cardiovascular disorders were reported in the secondary analysis Ⅱ, which focused on suspected cases only. This is most likely because the reporter was unsure whether the cardiovascular reactions were caused by the adverse effects of zolpidem and did not mark them as suspected drug reactions. The estimates from voluntary AE reports do not allow for the confirmation of causality or association. Additionally, insomnia itself is known to be a risk factor for heart failure or myocardial infarction (Sofi et al., 2014; Javaheri and Redline, 2017). Due to the inherent limitations of the data source, our results do not provide conclusive evidence on the relationship between zolpidem and cardiovascular disease and, if present, whether women are at higher risk of zolpidem-related cardiovascular disease. This results support the need for future research to test the hypotheses.

### Hyperkinesia, insomnia, and movement disorder

In our study, men were more likely to report insomnia, hyperkinesia, or aggressive reaction than women. Although the frequency and size of the difference are varied, these AEs seem to share a common possibility of being related to a rebound effect from zolpidem with a short half-life (Denise and Bocca, 2003; Ebert et al., 2006). While the rebound symptoms of non-benzodiazepine hypnotics are well established (Voshaar et al., 2004; Ebert et al., 2006), studies on gender difference is limited. In the *post hoc* clinical trial of chronic nightly zolpidem, there was no gender difference in rebound insomnia, which differed from the results of our study (Roehrs and Roth, 2016). Movement disorders, including hyperkinesia and extrapyramidal disorders, also showed a higher ROR in men. Future research is needed to determine whether men are at greater risk for these neurocognitive disorders.

In case of cognitive disorders or delirium, both primary and two secondary studies showed higher reporting risk in men. To determine whether there are gender-specific vulnerabilities in the cognitive function issues brought on by zolpidem use, more research may be required.

### Strengths and limitations

To our knowledge, this study is the first to investigate overall gender differences in the risk of reporting AEs of zolpidem using national voluntary AE reporting data. The use of vast amounts of national data over the last 5 years would have yielded reliable findings. Second, by comparing the RORs that were not dependent on the size of drug use and reporting behavior for each gender, we were able to make a reasonable comparison of gender differences in reporting risk. A number of studies have shown a higher frequency or rate of AEs reported in women (Lucca et al., 2017; de Vries et al., 2019). However, gender differences in the scale of drug users were not taken into account (Yu et al., 2016), and even if it is considered as the denominator, reporting behavior is regarded as gender-dependent. A study indicated that healthcare professionals more frequently reported AEs for women. Conversely, serious reports were more frequently reported for men, which was also supported by our findings (Holm et al., 2017).

Our study has several limitations. First, owing to the inherent limitations of data source and the signal detection methodology, causal inferences were not possible. This study underscores the need for future research to investigate potential factors such as concomitant medications, comorbidities, pharmacokinetics, dose variability, and alcohol intake that may contribute to gender disparities in adverse effects. Secondly, the data quality is inconsistent and underreporting is prevalent, especially concerning AEs like complex sleep behaviors, where concerns about discontinuing prescriptions or embarrassment may lead to underreporting. Thirdly, although the average daily prescription dose was similar for each gender, it remains unknown whether the dose variability affects the individual AEs signal discrepancies by gender. Furthermore, only 2,442 out of 11,341 reports contained dose information. Lastly, using data limited to Korean population makes it challenging to directly compare with studies involving other populations, particularly regarding genetic diversity and pharmacogenetics.

## Conclusion

The analysis of real-world data showed that reporting of AEs among zolpidem users was different by gender. This gender imbalance was pronounced in some of AEs such as parasomnia including somnambulism and cardiovascular disorders which is dominant in women, while cognitive disorders and insomnia are more frequent in men. Specifically, women demonstrated a greater vulnerability to somnambulism, which is a potentially severe adverse effect of zolpidem. The results support the need for more comprehensive clinical research on gender differences related to zolpidem in the future.

## Data Availability

Publicly available datasets were analyzed in this study. This data can be found here: https://open.drugsafe.or.kr/original/invitation.jsp.

## Abbreviations

ADR, adverse drug reaction; AE, Adverse event; ATC, Anatomical Therapeutic Chemical Classification System; BCPNN, Bayesian confidence propagation neural network; GABAA, γ-Aminobutyric acid type A; IC, Information component; IR, immediate release; KAERS, Korea voluntary adverse drug events reporting system; KIDS, Korea Institute of Drug Safety and Risk Management; MR, modified release; PRR, proportional reporting ratio; PT, preferred term; ROR, reporting odds ratio; WHO-ART, World Health Organization Adverse Reaction Terminology.
