# Supplementary material for: Gender differences in spontaneous adverse event reports associated with zolpidem in South Korea, 2015–2019
Source: Front Pharmacol. 2023 Nov 9;14:1256245. doi: 10.3389/fphar.2023.1256245 (PMC10665515; doi:10.3389/fphar.2023.1256245)
Supplement: Supplementary file 1 [file Table1.DOCX]

Supplementary table 1. Signals of adverse events related to zolpidem detected by data mining in total study population and by gender, 2015-2019

| Adverse event | No. of cases in total | PRR  in total | ROR  in toal | IC (95% LCI)  In total | No. of cases in women | ROR in women | No. of cases in men | ROR in men |
| --- | --- | --- | --- | --- | --- | --- | --- | --- |
| Delirium | 1024 | 59.06 | 64.82 | 5.54 | 333 | 55.88 | 691 | 67.7 |
| Insomnia | 562 | 4.30 | 4.47 | 1.96 | 309 | 4.16 | 253 | 5.17 |
| Confusion | 337 | 50.75 | 52.28 | 5.28 | 123 | 57.03 | 214 | 47.08 |
| Stupor | 148 | 8.21 | 8.31 | 2.77 | 68 | 7.42 | 80 | 9.27 |
| Hyperkinesia | 147 | 23.50 | 23.79 | 4.22 | 52 | 17.31 | 95 | 29.57 |
| Hallucination | 141 | 24.20 | 24.49 | 4.25 | 70 | 25.1 | 71 | 23.63 |
| Amnesia | 119 | 20.32 | 20.53 | 4.00 | 60 | 20.28 | 59 | 20.78 |
| Anxiety | 118 | 4.56 | 4.60 | 1.92 | 47 | 3.47 | 71 | 5.9 |
| Sleep Disorder | 91 | 11.50 | 11.59 | 3.18 | 42 | 9.76 | 49 | 14.06 |
| Depression | 61 | 3.44 | 3.45 | 1.42 | 24 | 2.37 | 37 | 5.09 |
| Somnambulism | 56 | 111.20 | 111.75 | 5.95 | 42 | 150.85 | 14 | 64.52 |
| Suicide Attempt | 54 | 17.39 | 17.47 | 3.67 | 29 | 20.23 | 25 | 14.77 |
| Thinking Abnormal | 52 | 14.24 | 14.30 | 3.39 | 24 | 13.63 | 28 | 14.75 |
| Paroniria | 49 | 31.11 | 31.24 | 4.42 | 31 | 40.41 | 18 | 22.23 |
| Agitation | 49 | 8.97 | 9.00 | 2.74 | 20 | 7.83 | 29 | 9.85 |
| Nervousness | 40 | 4.82 | 4.83 | 1.83 | 16 | 4.34 | 24 | 5.09 |
| Speech Disorder | 35 | 5.10 | 5.11 | 1.89 | 21 | 7.18 | 14 | 3.44 |
| Drug Dependence | 31 | 22.70 | 22.76 | 3.92 | 18 | 23.41 | 13 | 22.6 |
| Extrapyramidal Disorder | 27 | 3.19 | 3.20 | 1.17 | 11 | 3.06 | 16 | 3.18 |
| Dyskinesia | 22 | 4.97 | 4.98 | 1.75 | 11 | 4.74 | 11 | 5.28 |
| Gait Abnormal | 22 | 3.97 | 3.98 | 1.43 | 12 | 4.44 | 10 | 3.5 |
| Appetite Increased | 22 | 3.26 | 3.27 | 1.15 | 17 | 4.21 | 5 | 1.97 |
| Hypocalcaemia | 21 | 2.07 | 2.08 | 0.50 | 11 | 2.46 | 10 | 1.72 |
| Pulmonary Infiltration | 16 | 3.74 | 3.75 | 1.27 | 4 | 2.04 | 12 | 5.09 |
| Fall | 16 | 2.27 | 2.27 | 0.56 | 11 | 2.65 | 5 | 1.81 |
| Herpes Simplex | 16 | 2.24 | 2.24 | 0.54 | 8 | 2.4 | 8 | 2.06 |
| Infection | 15 | 2.10 | 2.10 | 0.43 | 5 | 1.53 | 10 | 2.53 |
| Xerophthalmia | 14 | 2.16 | 2.17 | 0.46 | 7 | 1.86 | 7 | 2.71 |
| Aggressive Reaction | 13 | 5.66 | 5.66 | 1.80 | 2 | 1.9 | 11 | 8.66 |
| Neuralgia | 13 | 3.30 | 3.30 | 1.04 | 11 | 5.19 | 2 | 1.11 |
| Infusion Site Reaction | 13 | 2.07 | 2.08 | 0.38 | 10 | 3.37 | 3 | 0.89 |
| Delusion | 12 | 10.56 | 10.57 | 2.65 | 7 | 13.79 | 5 | 7.75 |
| Aphasia | 12 | 4.69 | 4.69 | 1.52 | 7 | 6.38 | 5 | 3.31 |
| Tooth Ache | 12 | 2.42 | 2.42 | 0.58 | 4 | 2.1 | 8 | 2.49 |
| Psychosis | 11 | 14.69 | 14.70 | 3.08 | 7 | 27.9 | 4 | 7.56 |
| Anal Ulcer | 11 | 11.28 | 11.29 | 2.72 | 11 | 13.82 | . | . |
| Cerebrovascular Disorder | 11 | 7.83 | 7.84 | 2.22 | 2 | 7.87 | 9 | 7.14 |
| Tachycardia Supraventricular | 11 | 3.28 | 3.28 | 0.99 | 2 | 1.69 | 9 | 3.91 |
| Adrenal Insufficiency | 11 | 2.50 | 2.50 | 0.60 | 1 | 0.79 | 10 | 2.98 |
| Cognitive Disorders | 10 | 7.48 | 7.49 | 2.13 | 5 | 9.57 | 5 | 5.87 |
| Respiratory Disorder | 10 | 2.34 | 2.34 | 0.48 | 4 | 2.39 | 6 | 2.2 |
| Personality Disorder | 9 | 12.85 | 12.86 | 2.84 | 7 | 21.04 | 2 | 5.36 |
| Hepatic Cirrhosis | 9 | 6.18 | 6.19 | 1.83 | . | . | 9 | 7.1 |
| Post-Operative Haemorrhage | 9 | 3.34 | 3.34 | 0.96 | 2 | 1.97 | 7 | 3.95 |
| Inappropriate Schedule Of Drug Administration | 9 | 2.12 | 2.12 | 0.31 | 1 | 0.47 | 8 | 3.81 |
| Sleep Apnoea | 8 | 7.33 | 7.34 | 2.04 | 1 | 3.11 | 7 | 8.48 |
| Tachycardia Ventricular | 8 | 7.31 | 7.32 | 2.03 | 2 | 11.23 | 6 | 5.96 |
| Schizophrenic Reaction | 8 | 5.15 | 5.16 | 1.54 | 7 | 7.4 | 1 | 1.76 |
| Migraine | 8 | 3.06 | 3.07 | 0.81 | 6 | 3.91 | 2 | 1.95 |
| Dementia | 8 | 2.08 | 2.08 | 0.25 | 2 | 1.24 | 6 | 2.58 |
| Bowel Motility Disorder | 7 | 10.38 | 10.38 | 2.48 | 7 | 11.76 | . | . |
| Acrodynia | 7 | 9.55 | 9.56 | 2.37 | . | . | 7 | 13.91 |
| Emotional Lability | 7 | 4.15 | 4.16 | 1.20 | 3 | 5.16 | 4 | 3.42 |
| Lactation Nonpuerperal | 7 | 2.83 | 2.83 | 0.66 | 7 | 3.23 | . | . |
| Herpes Nos | 7 | 2.46 | 2.46 | 0.46 | 3 | 3.72 | 4 | 1.82 |
| Drug Abuse | 6 | 40.41 | 40.43 | 4.20 | 2 | 25.37 | 4 | 58.11 |
| Therapeutic Response Increased | 6 | 7.65 | 7.65 | 2.02 | 5 | 8.52 | 1 | 6.57 |
| Vein Varicose | 6 | 4.21 | 4.21 | 1.18 | . | . | 6 | 4.59 |
| Amenorrhoea | 6 | 4.15 | 4.15 | 1.16 | 6 | 4.59 | . | . |
| Dreaming Abnormal | 5 | 9.99 | 10.00 | 2.33 | 3 | 14.89 | 2 | 6.42 |
| Thrombosis Coronary | 5 | 4.01 | 4.01 | 1.06 | 4 | 12.34 | 1 | 1 |
| Bladder Carcinoma | 5 | 3.79 | 3.79 | 0.98 | 2 | 13.17 | 3 | 2.32 |
| Abrasion Nos | 5 | 3.28 | 3.28 | 0.78 | 3 | 4.59 | 2 | 2.22 |
| Heart Disorder | 5 | 3.25 | 3.25 | 0.76 | 1 | 1.74 | 4 | 3.94 |
| Respiratory Depression | 5 | 3.03 | 3.03 | 0.66 | 3 | 8.93 | 2 | 1.39 |
| Tooth Caries | 5 | 2.96 | 2.96 | 0.63 | 3 | 3.92 | 2 | 2.11 |
| Diplopia | 5 | 2.79 | 2.79 | 0.55 | 3 | 2.88 | 2 | 2.77 |
| Tolerance | 4 | 23.83 | 23.84 | 3.42 | 4 | 54.81 | . | . |
| Hepatitis Cholestatic | 4 | 11.27 | 11.27 | 2.44 | . | . | 4 | 14.72 |
| Oculomotor Nerve Paralysis | 4 | 10.24 | 10.24 | 2.31 | . | . | 4 | 13.63 |
| Artery Malformation | 4 | 9.61 | 9.61 | 2.22 | . | . | 4 | 9.05 |
| Skin Depigmentation | 4 | 8.92 | 8.92 | 2.12 | . | . | 4 | 10.32 |
| Myocarditis | 4 | 7.65 | 7.65 | 1.91 | 4 | 9.26 | . | . |
| Medication Error Related Problems | 4 | 7.00 | 7.00 | 1.79 | 3 | 11.68 | 1 | 3.1 |
| Unexpected Therapeutic Effect | 4 | 6.02 | 6.02 | 1.57 | 2 | 4.6 | 2 | 9.68 |
| Diverticulitis | 4 | 5.74 | 5.74 | 1.51 | 3 | 9.51 | 1 | 2.55 |
| Periodontal Disorders | 4 | 4.24 | 4.25 | 1.09 | 3 | 6.34 | 1 | 2.12 |
| Atherosclerosis | 4 | 3.43 | 3.43 | 0.79 | 4 | 8.46 | . | . |
| Otitis Externa | 4 | 3.40 | 3.41 | 0.77 | 2 | 2.91 | 2 | 4.28 |
| Otitis Media Chronic | 4 | 3.25 | 3.25 | 0.71 | 1 | 2.48 | 3 | 3.41 |
| Hemiparesis | 4 | 3.15 | 3.15 | 0.67 | 3 | 5.9 | 1 | 1.26 |
| Retinal Detachment | 4 | 3.13 | 3.13 | 0.66 | . | . | 4 | 3.49 |
| Decubitus Ulcer | 4 | 3.06 | 3.06 | 0.62 | . | . | 4 | 4.7 |
| Coma | 4 | 3.05 | 3.05 | 0.62 | 1 | 1.65 | 3 | 4.18 |
| Myopathy | 4 | 2.67 | 2.67 | 0.43 | 1 | 1.54 | 3 | 3.42 |
| Communication Disorder | 3 | 77.45 | 77.47 | 4.67 | 1 | 171.19 | 2 | 55.19 |
| Varicella | 3 | 10.33 | 10.33 | 2.25 | 3 | 22.34 | . | . |
| Haemangioma Acquired | 3 | 7.15 | 7.15 | 1.75 | . | . | 3 | 8.04 |
| Pain Axillary | 3 | 6.78 | 6.79 | 1.68 | 3 | 8.22 | . | . |
| Ecg Abnormal Specific | 3 | 4.89 | 4.89 | 1.22 | 1 | 5.71 | 2 | 4.24 |
| Wound Dehiscence | 3 | 4.87 | 4.87 | 1.21 | 3 | 10.27 | . | . |
| Menopausal Symptoms | 3 | 4.06 | 4.06 | 0.96 | 1 | 1.96 | 2 | 10.22 |
| Oculogyric Crisis | 3 | 3.59 | 3.59 | 0.79 | 2 | 4.79 | 1 | 2.38 |
| Leukaemia Lymphocytic | 3 | 3.33 | 3.33 | 0.68 | 3 | 8.63 | . | . |

PRR, proportional reporting ratio; ROR, reporting odds ratio; IC 95% LCI, information component lower limit of 95% confidence interval.
